# Condensation sink of atmospheric vapors: the effect of vapor properties and the resulting uncertainties

**DOI:** 10.1039/d1ea00032b

**Published:** 2021-09-23

**Authors:** Santeri Tuovinen, Jenni Kontkanen, Runlong Cai, Markku Kulmala

**Affiliations:** Institute for Atmospheric and Earth System Research, University of Helsinki Helsinki 00014 Finland markku.kulmala@helsinki.fi; Joint International Research Laboratory of Atmospheric and Earth System Sciences, School of Atmospheric Sciences, Nanjing University Nanjing China; Aerosol and Haze Laboratory, Beijing Advanced Innovation Center for Soft Matter Sciences and Engineering, Beijing University of Chemical Technology (BUCT) Beijing China; Faculty of Geography, Lomonosov Moscow State University Moscow Russia

## Abstract

Aerosol particles affect the climate and human health. Thus, understanding and accurately quantifying the processes associated with secondary formation of aerosol particles is highly important. The loss rate of vapor to aerosol particles affects the mass balance of that vapor in the atmosphere. The condensation sink (CS) describes the condensation rate of vapor to particles while the effective condensation sink (CS_eff_) describes the loss rate including both condensation and evaporation of vapor. When the CS is determined, the mass accommodation coefficient (*α*) is usually assumed to be unity and the condensing vapor is often assumed to be sulfuric acid. In addition, evaporation is assumed to be negligible (CS_eff_ = CS) and the total loss rate of vapor is described by the CS. To study the possible uncertainties resulting from these assumptions, we investigate how vapor properties such as vapor mass and *α* affect the CS. In addition, the influence of evaporation on the CS_eff_ is evaluated. The CS and CS_eff_ are determined using particle number size distribution data from Beijing, China. Vapors are observed to have differing CSs depending on molecular mass and diffusivity volume and larger molecules are lost at a slower rate. If the condensing vapor is composed, for example, of oxidized organic molecules, which often have larger masses than sulfuric acid molecules, the CS is smaller than for pure sulfuric acid vapor. We find that if *α* is smaller than unity, the CS can be significantly overestimated if unity is assumed. Evaporation can significantly influence the CS_eff_ for volatile and semi-volatile vapors. Neglecting the evaporation may result in an overestimation of vapor loss rate and hence an underestimation of the fraction of vapor molecules that is left to form clusters.

## Abstract 2

The condensation sink depends on vapor properties and thus inaccurate assumptions about them can lead to uncertainties in the descriptions of vapor mass balance or cluster formation.

## Graphical abstract

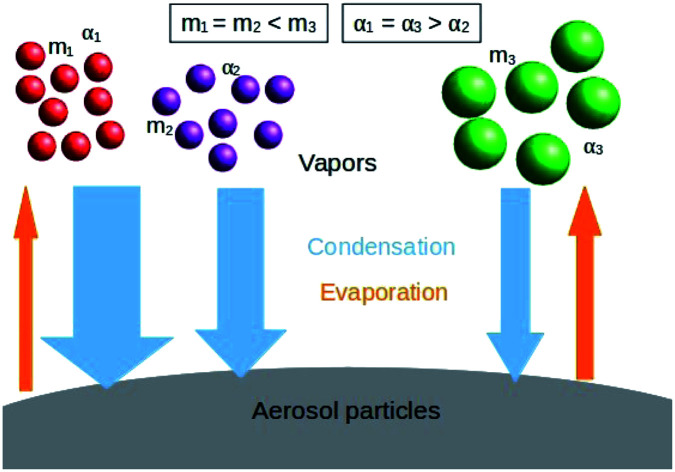

**Environmental significance**

The condensation sink (CS) affects the mass balance of atmospheric vapors and it can also influence the number of vapor molecules that form molecular clusters. These clusters can, if the conditions are suitable, grow to aerosol particles in a phenomenon called atmospheric New Particle Formation (NPF). Atmospheric NPF can influence climate and potentially also air quality. The CS is often determined making multiple assumptions and we evaluate the uncertainty arising from these assumptions. Our results can be applied to determine the CS more accurately for example in aerosol models, leading to a more comprehensive understanding of NPF.

## 1 Introduction

New Particle Formation (NPF), the formation and following growth of aerosol particles from atmospheric vapors,^1^ is globally a major source of atmospheric aerosol particles.^1–4^ Atmospheric aerosol particles have many effects both on climate^2,4–6^ and human health.^2,7^ For example, exposure to aerosol particles is associated with cardiovascular and respiratory diseases and leads to an increase in premature mortality.^7^ To improve the understanding of impacts of NPF, the processes affecting NPF need to be well understood.

For NPF to occur, the availability of atmospheric vapors is essential and thus one of the important parameters for understanding NPF is the condensation sink (CS).^8–10^ The CS, characterizing the loss rate of atmospheric vapor to aerosol particles, is widely used when studying aerosol dynamics.^11^ The CS may strongly affect the concentrations of atmospheric vapors and it can, for example, be used in estimating the source rates of atmospheric vapors, or in proxies for their concentrations.^12,13^ The CS is also important for understanding the mass balance of vapors in chamber studies.^14^ In addition, the coagulation sink (CoagS), the loss rate of small aerosol particles to larger aerosol particles, can be approximated using the CS.^15^

The CS depends, in addition to the number size distribution of aerosol particles, on environmental properties such as temperature and atmospheric pressure, and, more significantly, on vapor properties. The vapor properties include the molecular mass and mass accommodation coefficient, which has also been called the sticking probability.^16^ Several previous studies indicate that the mass accommodation coefficient is very likely close or equal to unity.^17–20^ However, there is still no consensus on values of the mass accommodation coefficient for atmospherically relevant vapors on aerosol particles in different environments.^21,22^ Because of this element of uncertainty, it is relevant to consider how much the CS could be reduced if the mass accommodation coefficient differs from unity.

The CS is often determined for a vapor consisting of sulfuric acid molecules, due to the key role of sulfuric acid in the formation of new aerosol particles.^23–25^ Sulfuric acid in the atmosphere is also expected to often be present clustered with bases and water.^26–28^ In addition to sulfuric acid and sulfuric acid clusters, organic compounds can have an important role in NPF by participating in cluster formation or by contributing to the growth of freshly formed clusters.^29–31^ Because the CS depends on the properties of the vapor, the loss rate of atmospheric vapors is expected to vary based on the condensing vapor.

The CS, describing the condensation rate to aerosol particles, is determined assuming irreversible condensation,^9,11,13^ however, in addition to condensation, there can be evaporation of vapors from the surface of the aerosol particles.^9,32^ This can affect the net flux of vapor to the particles if evaporation is considerable and thus the CS might not describe the total net loss rate of vapor accurately. In the case of sulfuric acid, this effect of evaporation on the loss rate is likely negligible due to the presence of stabilizing base sources.^33–35^ However, for atmospheric organic vapors with higher volatilities, evaporation could be significant and affect their loss rate.^14,36–38^ To describe the net loss rate accounting both for condensation and evaporation fluxes, we use the effective condensation sink (CS_eff_).

For NPF to occur, molecular clusters need to be formed at a sufficiently high rate. The fraction of vapor molecules that can form clusters is influenced by the loss rate of vapor to pre-existing particles. Thus, the overestimation of the CS and CS_eff_ can lead to inaccurate description of clustering.

The objective of this study is to provide a more comprehensive understanding of the condensation loss rate of atmospheric vapor to aerosol particles: (1) we investigate the dependency of the CS on vapor properties and compare values of the CS for different atmospherically relevant compounds. (2) We determine the dependency of the CS on the mass accommodation coefficient and (3) evaluate the effect of evaporation on the CS_eff_ of atmospheric vapors such as sulfuric acid and oxidized organic vapors. (4) Finally, we determine the effect of mass accommodation coefficient and non-negligible evaporation on the fractions of sulfuric acid molecules that are lost to pre-existing aerosol particles and that form clusters.

## 2 Methods

### 2.1 Condensation sink and effective condensation sink

The condensation sink (CS) was calculated using particle number size distribution1
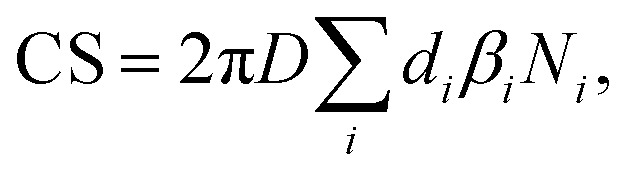
where *D* is the diffusion coefficient of the vapor, *β*_*i*_ is the Fuchs–Sutugin correction coefficient, *d*_*i*_ is the diameter of particles *i* and *N*_*i*_ is their number concentration.^8,9^ It was assumed that the condensation of vapor to aerosol particles can be accurately approximated by condensation of vapor on stationary particles. This assumption is valid when particles are much larger than vapor molecules and their diffusion is orders of magnitude slower than that of vapor molecules. The vapor diffusion coefficient *D* is obtained from2
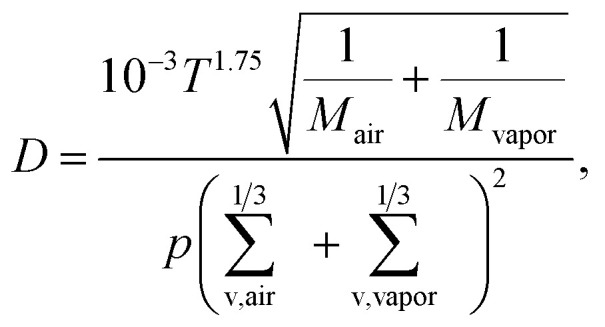
where *T* is the temperature, *p* is the atmospheric pressure, *M*_air_ and *M*_vapor_ are molar masses of air molecules and vapor molecules correspondingly and *Σ*_v,air_ and *Σ*_v,vapor_ are their diffusivity volumes.^33,34^ The Fuchs–Sutugin correction coefficient was calculated as3
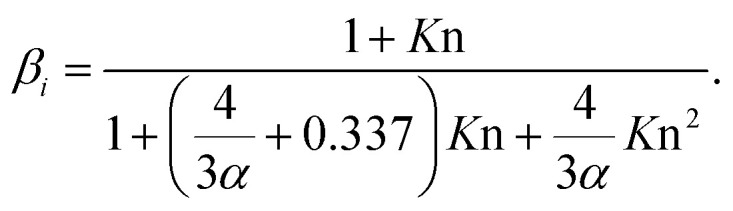
Here *K*n is the Knudsen number, *K*n = 2*λ*/*d*_*i*_, where *λ* is the mean free path of vapor molecules. *α* is the mass accommodation coefficient and it describes the probability that a vapor molecule or cluster sticks to a particle.^16^

The CS describes the flux of vapor to particles due to condensation. However, in addition there can be a non-negligible flux of the same vapor from particles due to evaporation. The vapor mass balance equation is^10^4
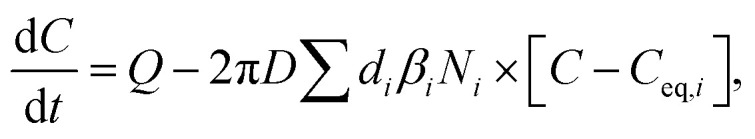
where *C* is the vapor number concentration, *t* is time, *Q* is the vapor source rate and *C*_eq,*i*_ is the vapor equilibrium concentration with respect to particles of size *d*_*i*_. *C*_eq,*i*_ was approximated using the Kelvin equation5
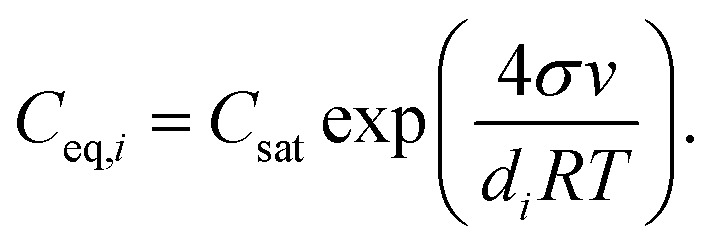
Here *C*_sat_ is the saturation vapor concentration with respect to a planar surface, *σ* is the surface tension of the vapor–liquid interface, *v* is the molar volume of the vapor, *d*_*i*_ is the diameter of the particle and *R* is the gas constant.^35^ We have defined the total loss rate of vapor to particles accounting for both condensation and evaporation fluxes as the effective condensation sink6
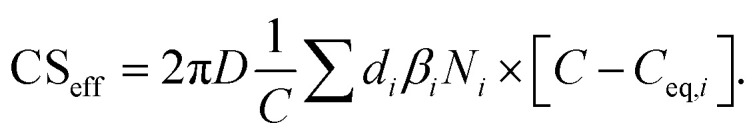


Using CS_eff_, the vapor mass balance equation is7
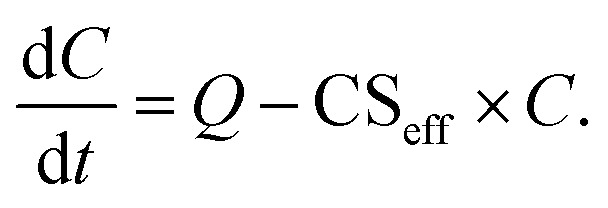


If the influence of Kelvin effect can be assumed to be negligible across the particle number size distribution, *C*_eq,*i*_ ≈ *C*_sat_ and8
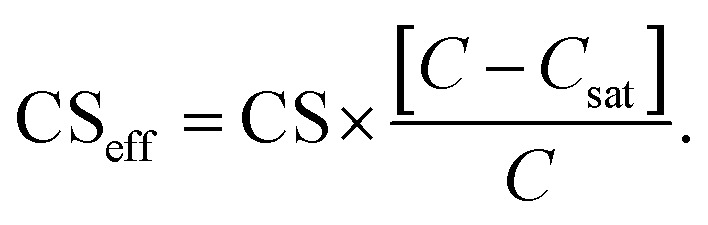


If *C*_sat_ approaches zero, evaporation flux is negligible, the CS_eff_ approaches the CS and the total loss rate of vapor to particles is only affected by the condensation flux described by the CS. In this work we used the CS_eff_ to describe the total vapor loss rate when evaporation is not assumed to be negligible while the CS was used when evaporation was not considered or when we assumed it to be negligible (CS_eff_ = CS).

### 2.2 Particle number size distribution

To calculate the CS and CS_eff_, we used particle number size distribution data measured at the station of Beijing University of Chemical Technology (39°56′31′′N, 116°17′50′′E, Beijing). The number size distributions between 1 nm and 1 μm were measured with a Diethylene Glycol Scanning Mobility Particle Sizer^36^ and a custom-made Particle Size Distribution system^37^ between January 2018 and March 2019. In the Beijing measurements, the relative humidity (RH) of the aerosol sample was conditioned to be below 40% by using a Nafion dryer. The hygroscopic growth factor for accumulation mode particles in Beijing has been estimated to be 1.3 at RH = 90%.^38^ Thus, for our case the error resulting from neglecting hygroscopic growth is likely small. However, it should be noted that depending on the composition of background particles hygroscopic growth can be significant and result in underestimation of the CS and CS_eff_ if it is neglected. The CS and CS_eff_ were calculated from median particle number size distributions between 9 and 12 am (local time, UTC + 8) from data for NPF events and non-NPF days separately. The day was classified as an NPF event day if a new particle mode below 25 nm appeared and growth of that mode was observed in the following hours.^39^ The days, on which there were clearly no NPF events, were classified as non-NPF days. More details on the particle size distribution measurements and NPF event classification are presented by Zhou *et al.*^40^

For comparison, we also calculated the CS from a median NPF event day particle number size distribution between January 2018 and March 2019 (9–12 am, UTC + 2) from Hyytiälä, Finland. The particle number size distribution data used to determine the median size distribution was measured at the Station for Measuring Forest Ecosystem-Atmosphere Relations (SMEAR) II (61°5′N, 24°170′E) using a twin DMPS (Mobility Particle Sizer) system.^41^ For more details on the measurements see *e.g.* Aalto *et al.*^41^

**Fig. 1:**
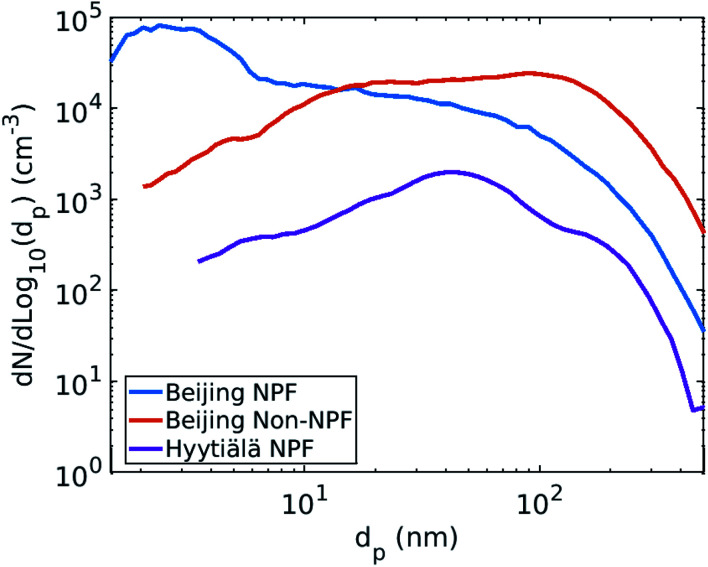
Median particle number size distribution in Beijing, China during NPF event days and non-NPF days, and in Hyytiälä, Finland during NPF event days (9–12 am) from January 2018 to March 2019.

The median particle number size distributions used in this work are presented in Fig. 1. To consider the influence of shape of the particle number size distribution on the CS and CS_eff_, three different particle number size distributions from two different locations were used. Only particles below 500 nm in diameter are considered to eliminate error resulting from differences in measurement set-ups between the two locations. In this work we did not investigate the dependency of the vapor loss on the particle number size distribution in more depth.

### 2.3 Investigated cases and system properties

We calculated the CS according to eqn (1). We investigated the sensitivity of the CS on the molecular mass (*m*) and diffusivity volume (*Σ*_v_) of the vapor and on the vapor diffusivity (*D*), which depends on the two aforementioned properties. To demonstrate the effect of vapor properties on the CS, we determined the CS for clusters of sulfuric acid and dimethylamine (DMA) or ammonia, and for the oxidation products of monoterpenes with chemical formulas of C_8–10_H_12–18_O_4–9_ (monomers) and C_16–20_H_24–36_O_8–14_ (dimers).^42^ In addition, a model oxidized organic molecule (OOM) was used to represent an average atmospheric organic molecule. Properties of model OOM were chosen based on the work by Ehn *et al.*^43^ In addition, vapors of oleic acid and C_5_H_10_O_5_, which is an oxidation product of isoprene, were used in Section 3.5. The compounds and their properties are presented in Table 1.

**Table 1:** Molecular mass (*m*), diffusivity volume (*Σ*_v_), surface tension (*γ*) and density (*ρ*) of compounds used in this work. Model OOM stands for the model oxidized organic molecule

Compound	*m* (u)	*Σ*_v_ (cm^3^)	*γ* (N m^−1^)	*ρ* (kg m^−3^)
Sulfuric acid (SA)	98.1	52.0	0.055	1830.0
DMA	45.1	52.5		
Ammonia (NH_3_)	18.0	11.5		
1 SA + 1 DMA	143.2	104.5	0.023	1500
2–5 SA + 0–4 DMA	196.2–670.9	104.0–470.0		
1–5 SA + 0–4 NH_3_	98.1–562.5	52.0–275.5		
C_8–10_H_12–18_O_4–9_	172.0–182.0	179.4–255.6	0.020	1500
C_16–20_H_24–36_O_8–14_	344.0–502.0	358.7–486.7	0.020	1500
Model OOM	325.0	300.0	0.020	1500
Oleic acid	282.5	377.0	0.033	895.0
C_5_H_10_O_5_	150.1	133.2	0.020	1500

We also investigated the dependency of the CS on the mass accommodation coefficient *α* (see eqn (3)). In the calculations, the value of *α* was assumed to be independent of the particle diameter. *α* is often assumed to be equal to unity and we estimated the resulting uncertainty if in some case this assumption is inaccurate.

We note that while we have investigated the dependency of vapor loss rates on *Σ*_v_, *m*, *D* and *α* using the CS, the same dependencies also apply to the total vapor loss rate, CS_eff_.

Finally, we investigated how much vapor evaporation can affect the vapor loss rate by determining the CS_eff_ according to eqn (6). Both sulfuric acid vapor and model OOM were used to calculate the CS_eff_. We divided OOMs based on their saturation concentrations to extremely low volatile organic compounds (ELVOCs), low volatile organic compounds (LVOCs) and semi-volatile organic compounds (SVOCs) using a classification based on previous research.^42,44,45^ For our model OOM if *N*_sat_ < 5.56 × 10^4^ cm^−3^ it was classified as ELVOC, and if *N*_sat_ > 5.56 × 10^8^ cm^−3^ it was classified as SVOC. If *N*_sat_ was in between these two limits, model OOM was classified as LVOCs.

To obtain saturation vapor pressures in a system consisting of sulfuric acid and ammonia, we used the E-AIM model (version II; http://www.aim.env.uea.ac.uk, last access: 30.07.2021). For more details on the model see *e.g.* Clegg *et al.*^46^ The sum of sulfuric acid concentrations in the gaseous and aerosol phase was assumed to be equal to 4 × 10^−8^ mol m^−3^ (corresponding to 3.9 μg m^−3^). Relative humidities RH = 10%, 30%, 50% and 70%, and temperatures *T* = 285 K and 298 K were used. It should be noted that values of vapor pressure were not sensitive to the assumed concentration of sulfuric acid. For example, setting the total concentration of sulfuric acid to 1 × 10^−7^ mol m^−3^ instead would not change our results significantly.

It should be noted that atmospheric aerosol particles consist of mixtures of different chemical compounds, which affect the *C*_sat_ of the condensing species. The interactions of the molecules of the studied condensing species and the molecules of other compounds in the particle phase vary depending on the identity of the compounds, which can be accounted for by using the activity coefficient.^35^ In this work, we do not explicitly include the activity coefficient in eqn (5). For model OOM, we do not consider the effect of activity. For the system with sulfuric acid and ammonia, *C*_sat_ obtained from the E-AIM model accounts for activity in a simplified system of water, ammonia and sulfuric acid. However, in a real atmospheric system the chemical composition of particles varies resulting in varying activities and *C*_sat_ values.

We used atmospheric pressure *P* = 101 325 Pa in all of our calculations. Temperature *T* = 285 K was used if not otherwise stated. Choice of *T* and *P* had only a minor influence on our results.

### 2.4 Losses of sulfuric acid monomers

To illustrate the effect of changes in the CS on the dynamics of atmospheric systems, we determined the fractions of sulfuric acid monomers lost by condensation to aerosol particles and by clustering of vapor monomers at different CS values, assuming that these are the only loss processes of sulfuric acid monomers and there is for example no losses due to deposition or chemical reactions.

We used the kinetic model presented by Cai *et al.*^47^ The losses of sulfuric acid monomers due to condensation are determined as

9 *L*_CS_ = *C*_SA_(CS_SA_(1 − *η*) + CS_1SA+DMA_*η*)

where *C*_SA_ is the total concentration of sulfuric acid monomers, CS_SA_ is the CS of sulfuric acid molecules while CS_1SA+1DMA_ is the CS of a cluster consisting of sulfuric acid molecules and DMA molecules. *η* is the ratio of sulfuric acid clusters of one sulfuric acid molecule and one base molecule to pure sulfuric acid molecules. It is10
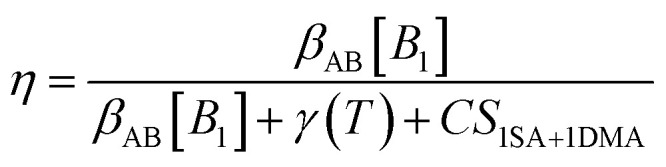
[*B*_1_] is the concentration of bases. We assumed [*B*_1_] to correspond to the concentration of DMA and [*B*_1_] = 2.7 ppt. The collision coefficient between *A*, in this case sulfuric acid, and *B* is *β*_AB_ = 4.58 × 10 cm^−3^ s^−1^ × 2.29, where 2.29 is the van der Waals enhancement factor.^48,49^ Evaporation coefficient is *γ*(285 K) = 0.092 s^−1^.

At the kinetic limit the losses due to clustering are

11 *L*_KL_ = *β*_11_[*A*_1,tot_]^2^

where [*A*_1,tot_] is the total sulfuric acid monomer concentration, and [*A*_1,tot_] = *C*_SA_. *β*_11_ is the coagulation coefficient between sulfuric acid monomers, and *β*_11_ = 4.8 × 10 cm^−3^ s^−1^ × 2.29. The real losses due to clustering are

12 *L* = *L*_KL_*η*(2 − *η*).

## 3 Results

### 3.1 Dependency of the CS on vapor properties

**Fig. 2:**
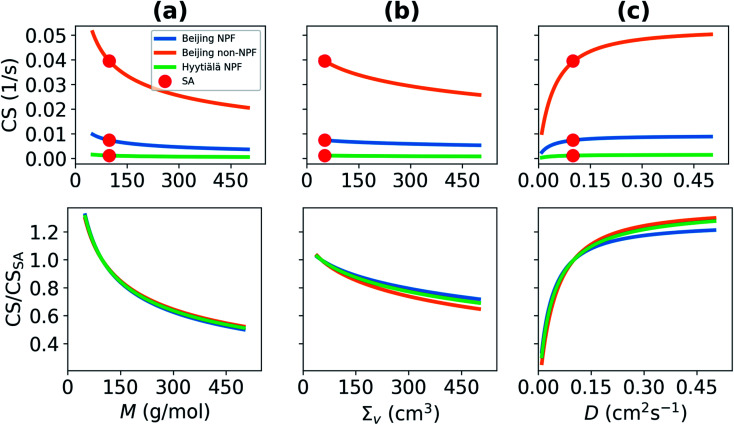
Condensation sink (CS), and the ratio of the CS and CS of sulfuric acid monomers (SA), as a function of the (a) molar mass (*M*), (b) diffusivity volume (*Σ*_v_), and (c) diffusion coefficient (*D*). Other properties correspond to those of SA. The CS has been determined using the median NPF and non-NPF particle number size distribution from Beijing, China, and median NPF size distribution from Hyytiälä, Finland (see Section 2.2). The values corresponding to those of SA are shown.

The condensation loss rate of vapor depends on its properties. We investigate the sensitivity of the condensation sink (CS) on the molar mass (*M*), diffusivity volume (*Σ*_v_) and on the diffusion coefficient (*D*), which depends on the former two quantities (Fig. 2). In all cases other properties are assumed to correspond to properties of sulfuric acid vapor (*M* = 98.08 g mol^−1^, *Σ*_v_ = 51.96 cm^3^). We used the median particle number size distributions of NPF days in Beijing and in Hyytiälä, and the median particle number size distribution of non-NPF days in Beijing to determine CS. With increasing *M*, the CS decreases (Fig. 2a). If *M* increases to 300 g mol^−1^, or more, from *M* of the sulfuric acid monomer, Beijing median NPF day CS decreases by more than 37%. This corresponds to a CS < 0.0046 s^−1^ instead of a CS = 0.0074 s^−1^. *M* and *Σ*_v_ are both properties related to the size and composition of a molecule or a cluster and if *M* increases, in most cases *Σ*_v_ also changes. However, in this study, we studied the dependency on *M* and *Σ*_v_ separately. The CS is shown to also decrease with increasing *Σ*_v_ (Fig. 2b). If *Σ*_v_ > 300 cm^3^, CS < 0.006 s^−1^, which means that the CS is decreased by around 20% compared to the CS for sulfuric acid. If *M* and *Σ*_v_ of vapor increase, the diffusion of vapor molecules becomes slower and *D* decreases. This results in decreasing CS (Fig. 2c).

We also see from Fig. 2 that while the choice of particle number size distribution has a large effect on the absolute values of the CS, it has only a minor effect on the behavior of the CS as a function of *M*, *Σ*_v_ and *D*. At the most, the ratio of the CS and CS_SA_ differs by less than 10% between the three particle number size distributions. Because of this, we do not include the non-NPF Beijing and NPF Hyytiälä size distributions in further analysis of the CS for different atmospherically relevant compounds (Section 3.2).

Overall, these results illustrate that the CS can get significantly different values depending on vapor properties, and thus inaccurate assumptions about properties of the condensing vapor can lead to significant error in CS. In practice, if the vapor molecule has a larger molecular mass than the sulfuric acid monomer, which is for example the case for many organic vapors, the corresponding CS will be significantly lower than for the CS for sulfuric acid monomers (see Section 3.2). If a vapor has lower molecular mass than sulfuric acid, its CS will be higher than the CS for sulfuric acid.

### 3.2 CS of different atmospheric vapors and clusters

Atmospheric sulfuric acid can be present in clusters consisting of sulfuric acid and other compounds such as dimethylamine (DMA) or ammonia.^24,26,27,50,51^ The CS of these clusters is different from that of pure sulfuric acid molecules due to their larger mass. For example, if a major fraction of sulfuric acid monomers is present in clusters with DMA, the loss rate of sulfuric acid monomers is decreased as a result.

**Fig. 3:**
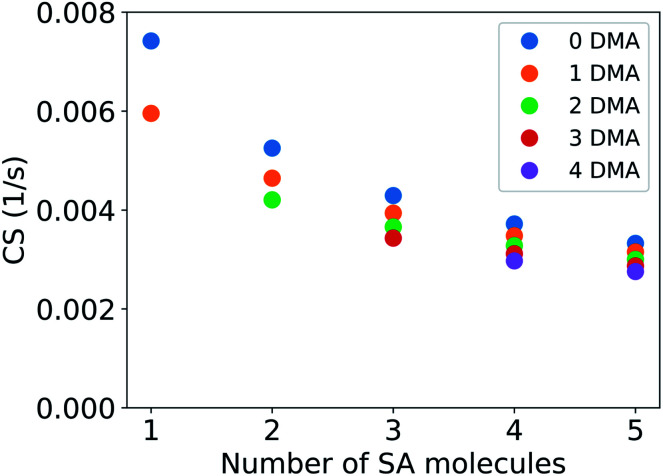
CS for clusters of sulfuric acid (SA) and dimethylamine (DMA) as a function of the number of SA molecules. The number of DMA molecules is shown with color. The CS has been determined using particle number size distribution for the median NPF day from Beijing, China (see Section 2.2).

Fig. 3 shows the CS for clusters composed of sulfuric acid and DMA. In the results presented here the CS has been determined using the median NPF day particle number size distribution for Beijing, China. The mass accommodation coefficient (*α*) is assumed to be equal to unity and evaporation of vapor from particles is assumed to be negligible. A cluster of two sulfuric acid molecules and one DMA has a CS of 0.0046 s^−1^, which is 62% of the loss rate for the sulfuric acid monomer. A large cluster composed of five sulfuric acid molecules and four DMA has a CS of 0.0028 s^−1^, which is 38% of the CS for the sulfuric acid monomer. The CS of clusters with one to five sulfuric acid molecules and one to four DMA molecules varies between 19% and 38% of the loss rate for pure sulfuric acid monomers. As discussed in Section 2.1, the CS decreases with increasing molecular number due to increasing mass and diffusivity volume of the cluster. The addition of a sulfuric acid molecule to the cluster changes the CS more compared to the addition of a DMA due to the larger size of the molecules. In the atmosphere sulfuric acid–DMA clusters may also contain water,^28^ but the addition of water should have relatively little effect on the value of the CS due to the small size of water molecules and is not considered in this study.

**Fig. 4:**
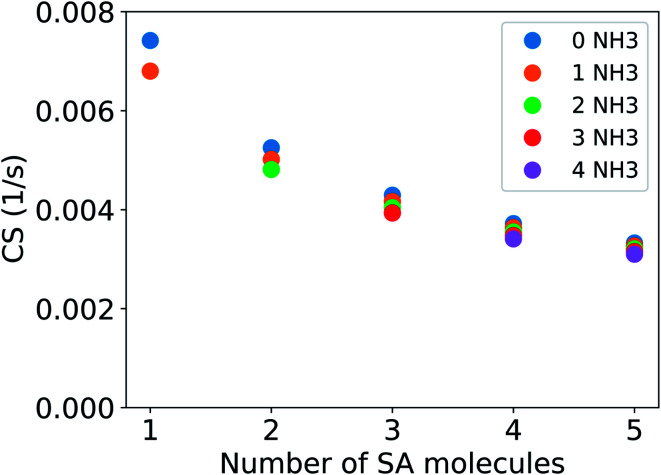
Condensation sink (CS) of clusters of sulfuric acid (SA) and ammonia (NH_3_) as a function of the number of SA molecules. The CS is determined using the median NPF day particle number size distribution from Beijing, China (see Section 2.2).

Fig. 4 shows the CS for clusters consisting of sulfuric acid and ammonia. The CS has been determined using the median NPF day particle number size distribution from Beijing, China. For a cluster with two sulfuric acid molecules and one ammonia molecule, CS = 0.0050 s^−1^, which is 68% of the CS for sulfuric acid monomers. If the vapor consists of clusters of one to five sulfuric acid and one to four ammonia molecules, the CS is between 0.0068 s^−1^ and 0.0031 s^−1^, *i.e.*, between 92% and 42% of the CS for pure sulfuric acid monomers. Similar to addition of DMA, addition of ammonia also decreases the CS. However, the change is smaller due to the smaller molecular size of ammonia compared to DMA.

If a major fraction of condensing vapor is expected to be other than sulfuric acid, this should to be taken into account when determining the CS. In Table 2 we have included the CS of some other atmospherically relevant compounds. The CS has been determined using median NPF day particle number size distribution from Beijing, China and we assumed *α* = 1 and that evaporation is negligible. The CS of the model OOM is 0.0038 s^−1^, which is 51% of the CS for sulfuric acid. For an oxidized organic monomer with 8–10 carbon atoms, the CS is between 0.0052 and 0.0041 s^−1^, depending on the composition. For the oxidized organic dimers the CS is 0.0037–0.0031 s^−1^. Due to their larger mass, many organic vapors are lost to particles at slower rates compared to sulfuric acid vapor.

**Table 2:** Condensation sinks (CS) of different atmospherically relevant compounds and clusters. Mass accommodation coefficient *α* is assumed to be equal to unity and evaporation is negligible. The CS has been determined using the particle number size distribution for the median NPF days from Beijing, China (see Section 2.2)

Compound	CS (s^−1^)
Sulfuric acid	7.4 × 10^−3^
(1–5) sulfuric acid + (0–4) DMA	7.4 × 10^−3^ – 2.8 × 10^−3^
(1–5) sulfuric acid + (0–4) ammonia	7.5 × 10^−3^ – 3.1 × 10^−3^
C_8–10_H_12–18_O_4–9_	5.2 × 10^−3^ − 4.1 × 10^−3^
C_16–20_H_24–36_O_8–14_	3.7 × 10^−3^ – 3.1 × 10^−3^
Model OOM	3.8 × 10^−3^

**Fig. 5:**
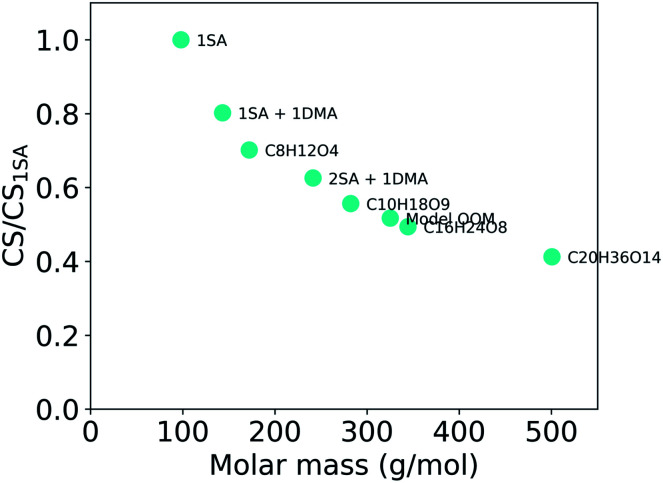
Ratio of the condensation sink (CS) for some atmospherically relevant compounds and for sulfuric acid (SA) as a function of molar mass. CS values have been calculated using the median NPF particle number size distribution from Beijing, China (see Section 2.2).

Overall, the CS strongly depends on the composition of the condensing vapor due to the differences in molecular mass and diffusivity. Fig. 5 presents the ratio of CS between different compounds, or clusters, and sulfuric acid as a function of molar mass. It shows a decreasing CS with increasing molar mass of these clusters and compounds. Because the CS varies with the properties of the vapor, the accuracy of assumptions about the properties of condensing vapor should be taken into consideration. For example, if the CS has been calculated for sulfuric acid monomers, that value only characterizes the sink of sulfuric acid monomers and not the sink of sulfuric acid clustered with water or base. In addition, various compounds other than sulfuric acid, such as OOMs, play important roles in atmospheric cluster formation and growth.^29–31,44^ All of these different vapors have differing loss rates. If the CS is for example used to evaluate the formation of atmospheric clusters, it is worth considering whether the CS should be determined taking into account sulfuric acid molecules, sulfuric acid clustered with bases or water or even OOMs.

One should note that the dependency of the CS on the vapor molecule size is analogous to the dependency of coagulation sink on the particle size. With increasing mass and particle size, the diffusivity of the particle decreases resulting in a lower coagulation sink.^8,15^ In this work we have treated small clusters similarly to vapor molecules.

### 3.3 Dependency of the CS on the mass accommodation coefficient

The value of the CS is a function of the mass accommodation coefficient *α* (see eqn (3)). The significance of this dependency is investigated in this section.

**Fig. 6:**
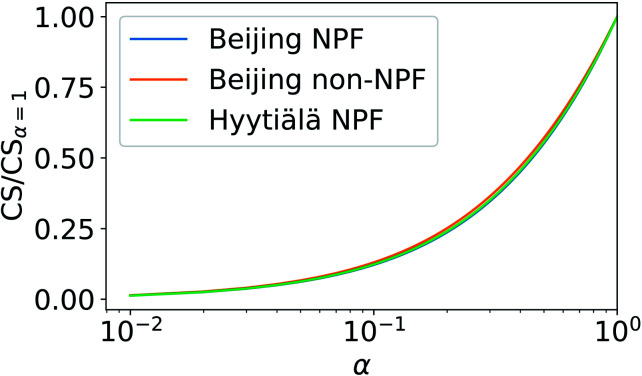
Ratio of the condensation sink (CS) of sulfuric acid (SA) with varying mass accommodation coefficients (*α*) to the CS assuming *α* = 1. CS values have been determined from three different particle number size distributions: median NPF size distributions from Beijing, China and Hyytiälä, Finland and median non-NPF size distribution from Beijing (see Section 2.2).

Fig. 6 shows the ratio of sulfuric acid CS with varying *α* to CS assuming *α* = 1. The CS is determined using median NPF size distributions from Beijing and Hyytiälä and median non-NPF size distribution from Beijing. CS/CS_*α*=1_ does not show major dependency on the used particle number size distribution. Thus, we only include the median NPF number size distribution from Beijing for further discussion.

**Fig. 7:**
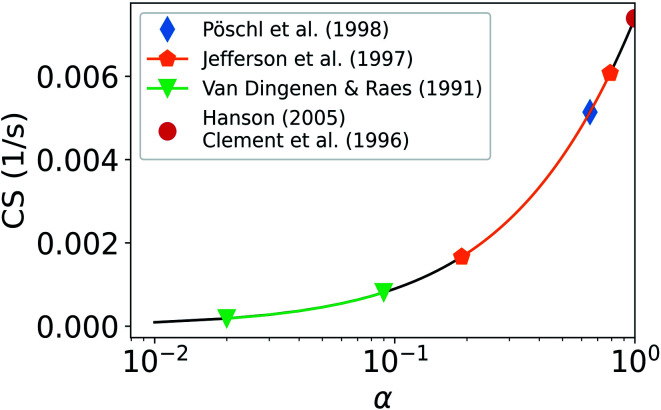
Condensation sink (CS) of sulfuric acid as a function of the mass accommodation coefficient *α*. Ranges and values from past studies for *α* have been marked and the CS has been determined using the median NPF day size distribution from Beijing, China (see Section 2.2).

Fig. 7 shows how the CS of sulfuric acid depends on *α*. If *α* = 1, CS = 0.0074 s^−1^. If *α* < 0.5 then the CS < 0.0041 s^−1^. Thus, if *α* < 0.5, the CS is below 56% of the corresponding value when *α* = 1. Therefore, if a significant fraction of collisions of vapor molecules onto particles does not result in the uptake of molecules, *i.e.*, *α* < 1, assuming *α* = 1 results in an overestimation of the vapor loss rate.

Fig. 7 also includes ranges for *α* as given by previous studies. Pöschl *et al.*^21^ reported the best fit for *α* of sulfuric acid vapor on aqueous sulfuric acid to be 0.65. If *α* = 0.65, CS = 0.0051 s^−1^, and if in this case we then assume *α* = 1, we will overestimate the CS by 45%. Van Dingenen and Raes^52^ reported *α* to be between 0.02 and 0.09 for sulfuric acid vapor on particles of sulfuric acid and water. Using this range of *α*, the calculated CS ranges from 1.8 × 10^−4^ s^−1^ to 8.1 × 10^−4^ s^−1^. Jefferson *et al.*^53^ reported *α* to be 0.79 for sulfuric acid on NaCl particles and between 0.19 and 0.31 for sulfuric acid on NaCl particles coated with stearic acid. If *α* is between 0.19 and 0.79, the CS is between 0.0017 s^−1^ and 0.0061 s^−1^. If in these cases *α* = 1 is assumed, the CS is overestimated by 335% to 21%. Clement *et al.*^17^ suggested that for sulfuric acid on atmospheric droplets, *α* should be near unity. Several more recent studies also suggest that *α* is close to, or equals, unity for sulfuric acid, organic vapors and water vapor.^18,54,55,56^ Roy *et al.*^22^ reported *α* of water vapor on organics to be 0.25 at 296 K and to decrease strongly with increasing temperature. Using predictions for *α* based on multiple previous studies, it becomes apparent that the CS differs strongly based on the assumed value of *α*.

We assumed the value of *α* to be constant across all the particle diameters. However, some studies suggest that *α* increases with increasing particle size.^57^ Since larger particles cause a larger sink compared to smaller particles, using *α* that increases with diameter instead of constant *α* would decrease the effect of non-unity *α* on CS.

Although several studies suggest that *α* is unity and that collisions between vapor molecules and aerosol particles stick, there is still no consensus. There is also no research on variance of *α* between different environments such as boreal forests and megacities. It is possible that atmospheric *α* could vary for example based on both vapor properties and the properties of background aerosol particles, such as their chemical composition. It is also possible that *α* varies depending on the size and atmospheric age of the aerosol particles. It is thus important to consider how the assumptions about *α* influence the value of CS.

In addition to being reduced due to collisions between vapor molecules and particles not sticking, the CS can be increased due to attractive intermolecular forces resulting in an increased number of collisions between molecules and particles.^48,49^ This enhancement resulting from van der Waals forces has been estimated to be 1.3 for collisions between sulfuric acid molecules and Aitken mode particles.^49^ Because the CS is dominated by Aitken and accumulation mode particles, we have chosen to neglect this enhancement. However, accounting for it would lead to a minor enhancement of the CS and would result in all CS and CS_eff_ values being slightly larger.

### 3.4 Influence of evaporation on the total vapor loss rate

#### 3.4.1 Evaporation effect on the loss rate of highly oxidized organic molecules

We investigate the effect of evaporation on the total loss rate of vapor on aerosol particles for different vapor concentrations (*C*) and saturation concentrations (*C*_sat_) using the effective condensation sink (CS_eff_, eqn (6)). The mass accommodation coefficient (*α*) is assumed to be equal to unity. CS_eff_ for different *C* and *C*_sat_ behaves in a similar way for all the investigated size distributions due to the influence of Kelvin effect being relatively minor. Here we present the CS_eff_ using only the median NPF day number size distribution from Beijing. We use the model OOM defined in Section 2.4 and vary *C*_sat_ and *C*_OOM_ to investigate the CS of OOMs. OOMs span a wide range of *C*_sat_ and we divided the model OOM into an extremely low volatile organic compound (ELVOC), low volatile organic compound (LVOC) or semi-volatile organic compound (SVOC) according to its *C*_sat_ (see Section 2.3).

**Fig. 8:**
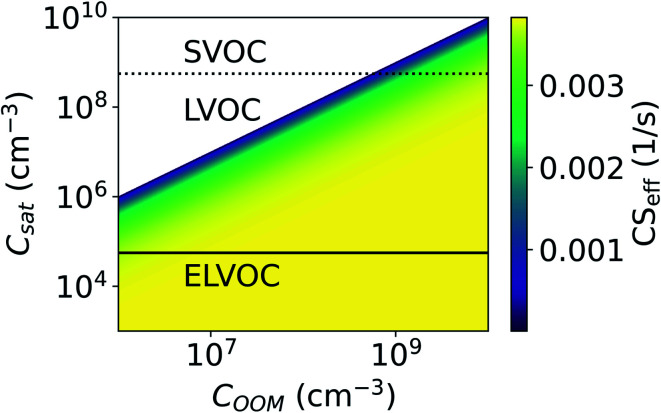
Effective condensation sink (CS_eff_) for a model oxidized organic molecule (OOM) as a function of vapor concentration (*C*_OOM_) and saturation concentration (*C*_sat_). CS_eff_ was determined using median NPF particle number size distribution from Beijing, China (see Section 2.2). Lines indicate whether OOM with certain *C*_sat_ would be categorized as an extremely low volatile organic compound (ELVOC), low volatile organic compound (LVOC) or semi-volatile organic compound (SVOC) (see Section 2.3). In the white area the flux resulting from evaporation is larger than the condensation flux.

Fig. 8 shows the CS as a function of *C*_OOM_ and *C*_sat_. With lower *C*_OOM_ and higher *C*_sat_, and thus lower saturation, the effect of evaporation becomes significant. Assuming *C*_OOM_ = 10^8^ cm^−3^ and *C*_sat_ = 10^7^ cm^−3^, CS_eff_ = 0.0034 s^−1^ and it is only 11% lower than CS. At *C*_OOM_ = 10^8^ cm^−3^ and *C*_sat_ = 5 × 10^7^ cm^−3^, CS = 0.0019 s^−1^, which is 50% of the CS. From Fig. 8 we can also see that for ELVOCs the effect of evaporation on the total vapor loss rate to particles is negligible for all *C*_OOM_ in the test range, but for SVOCs the effect of evaporation on the loss rate can be significant even for *C*_OOM_ up to 10^10^ cm^−3^. For both LVOCs and SVOCs in low *C*_OOM_ the evaporation flux can also be larger than the condensation flux and thus there is no condensation losses of vapors to particles. In these cases, the CS_eff_ acts as a rate constant of total evaporation of vapor from aerosol particles, increasing vapor concentration. We have chosen not to show negative CS_eff_ values to keep the focus of this study on vapor losses due to condensation.

We do not take into account in this study that the *C*_sat_ generally decreases with molecular mass. Therefore, we may overestimate the loss rate of high-volatility molecules such as SVOCs and higher volatility LVOCs whereas underestimate the sink of low-volatility molecules such as ELVOCs. In addition, we do not consider the possible variability in *C*_sat_ due to differences in particle chemical composition. However, it is clear that when either *C*_OOM_ is low or *C*_sat_ is high, resulting in low saturation ratio, evaporation considerably reduces the rate that the vapor is lost to the particles.

#### 3.4.2 Evaporation effect on sulfuric acid loss rate

**Fig. 9:**
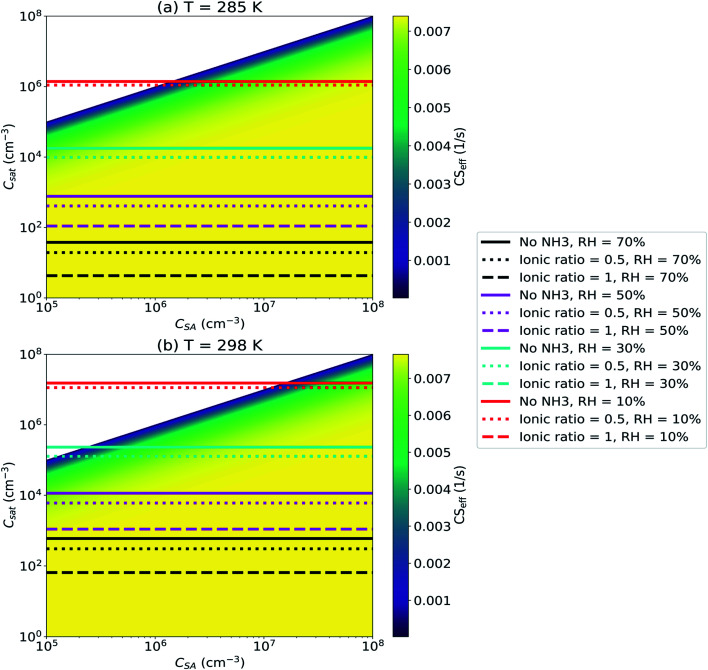
Sulfuric acid (SA) effective condensation sink (CS_eff_) as a function of vapor concentration (*C*_SA_) and saturation concentration (*C*_sat_) for temperatures of (a) *T* = 285 K and (b) *T* = 298 K. *C*_sat_ values determined with E-AIM for different ionic ratios [NH_4_^+^]/[SO_4_^2−^] and relative humidities (RH) have been marked. CS_eff_ was determined using median NPF particle number size distribution from Beijing, China (see Section 2.2).

Fig. 9 illustrates the effective condensation sink (CS_eff_) for sulfuric acid as a function of vapor concentration (*C*_SA_) and saturation concentration (*C*_sat_). The mass accommodation coefficient (*α*) is assumed to be equal to unity and CS_eff_ is determined using particle number size distribution for the median NPF day in Beijing. The results from the E-AIM model are included in Fig. 9 and they show the CS_eff_ for systems with three different ionic ratios of NH_4_^+^ and SO_4_^2−^ and four different relative humidities (RH). The ionic ratio [NH_4_^+^]/[SO_4_^2−^] corresponds to the ratio of ammonia and sulfuric acid in the system. Results for *T* = 285 K (Fig. 9a) and *T* = 298 K (Fig. 9b) are shown.

Fig. 9 shows that RH has a major influence on *C*_sat_ and evaporation only affects the CS_eff_ for low RH due to the large lowering effect of water on SA *C*_sat_. At *T* = 285 K, evaporation has any effect on the CS_eff_ only for RH as low as 10% and for a system with [NH_4_^+^]/[SO_4_^2−^] ≤ 0.5. In systems with higher RH evaporation has no significant effect on the CS_eff_. At *T* = 298 K, evaporation lowers the CS if [NH_4_^+^]/[SO_4_^2−^] ≤ 0.5, RH ≤ 30% and *C*_SA_ < 10^6^ cm^−3^. It is apparent that the CS_eff_ can be significantly affected by evaporation only if [NH_4_^+^]/[SO_4_^2−^] and RH are low and *T* is sufficiently high.

We note that we have only considered a system with sulfuric acid, water and ammonia and have not studied systems with other basic components. Saturation concentration of sulfuric acid depends not just on the concentration of basic components but also on the chemical species. Addition of other bases than ammonia such as DMA to the system changes *C*_sat_. DMA is a stronger base than ammonia and if the system includes DMA in addition to, or instead of, ammonia, *C*_sat_ is likely lower and evaporation has less effect on the CS_eff_.^58^ The studied system also includes only particles consisting of sulfuric acid, ammonia and water while real atmospheric aerosol particles have varying compositions and consist of a wide range of chemical compounds. Thus, in the atmosphere *C*_sat_ can vary while here we assume it to be constant.

It appears that in most atmospheric, especially urban, environments the sulfuric acid CS_eff_ is likely not affected by evaporation. Water by itself effectively lowers sulfuric acid *C*_sat_ and bases such as ammonia, or stronger bases such as DMA, additionally lower *C*_sat_ resulting in evaporation being negligible compared to condensation. However, it is still possible that in some environments with both low humidity and low concentrations of bases, the sulfuric acid CS_eff_ is significantly reduced due to non-negligible evaporation of sulfuric acid from aerosol particles.

### 3.5 Relevance of results and implications

**Fig. 10:**
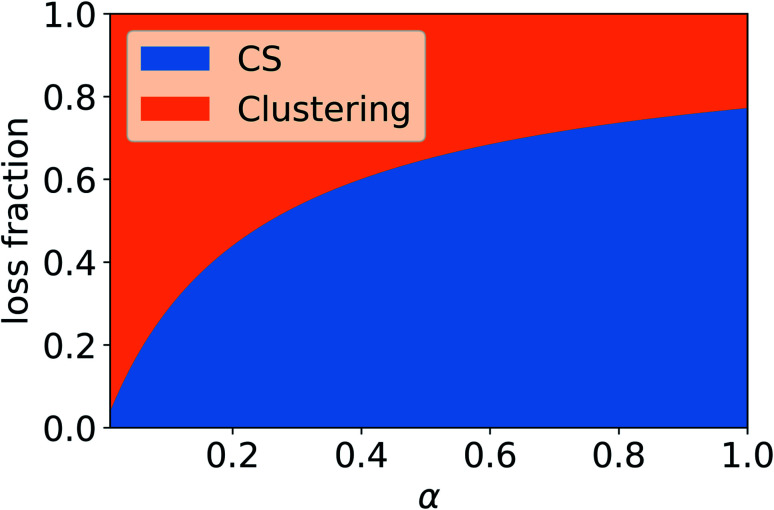
Sulfuric acid monomer loss fractions due to the condensation sink (CS) and cluster formation as a function of mass accommodation coefficient (*α*). Evaporation of sulfuric acid from aerosol particles is assumed to be negligible. The CS was determined using median NPF particle number size distribution from Beijing, China (see Section 2.2).

In addition to condensation on pre-existing particles, in the atmosphere vapor can be lost due to other processes, including the formation of molecular clusters, which may grow to larger particles. Therefore, if no other loss processes are considered, reduced vapor loss rate due to non-unity mass accommodation or significant evaporation from particles can increase the rate at which vapor forms molecular clusters. Fig. 10 shows the fraction of sulfuric acid (SA) monomers, including both SA molecules and clusters of one SA and DMA molecule, lost due to the CS and dimer formation as a function of the mass accommodation coefficient *α*. We can see that as *α* decreases, reducing the CS, the fraction of monomers lost to dimer formation increases. Compared to the case of *α* = 1, *α* has to be around 0.4 or less for the loss fraction due to the CS to decrease by more than 20%, If the CS is reduced due to non-unity *α*, or other factors such as evaporation affect the loss rate of vapor to particles, a larger number of monomers are potentially left to form dimers, which can then form larger clusters.

Table 3 summarizes the CS_eff_ of some atmospheric compounds, considering both previously reported values of *α* and the possibility of evaporation from particles using *C*_sat_ values based on previous studies. The CS_eff_ has been determined using the median NPF day particle number size distribution from Beijing, China. These compounds all have different CS_eff_ ranging from 0.0074 s^−1^ for pure sulfuric acid at *C*_vapor_ ≥ 1 × 10^5^ cm^−3^ to just 0.0018 s^−1^ at *C*_vapor_ = 2.5 × 10^10^ cm^−3^ for oleic acid. It is apparent that the vapors are lost to particles at different rates depending on their properties.

**Table 3:** Effective condensation sinks (CS_eff_) of atmospheric vapors determined using median NPF day particle number size distribution from Beijing, China (see Section 2.2). Mass accommodation coefficient (*α*) and saturation concentration (*C*_sat_) values have been chosen based on previous research aside from *C*_sat_ of pure sulfuric acid (SA), which was estimated based on the E-AIM model. Density of 1500 kg m^−3^ and surface tension of 0.02 N m^−1^ have been assumed for compounds C_*x*_H_*y*_O_*z*_. For SA and a cluster of SA and DMA, values using mean *α* based on previously reported values are presented (in parentheses) in addition to the commonly reported value of unity

Vapor	*α*	*C*_sat_ (cm^−3^)	*C*_vapor_ (cm^−3^)	CS_eff_ (s^−1^)	% of CS for SA (*α* = 1)	
SA (no bases, RH = 50%)	1.0 (0.44)	770.0	≥1 × 10^5^	0.0074 (0.0036)	100 (49)	17, 18, 21, 52 and 53
SA + DMA	1.0 (0.44)	0		0.0060 (0.0030)	81 (41)	26 and 50
C_10_H_18_O_9_	1.0	1.98 × 10^8^	1 × 10^8^	Evaporation > condensation	30	14, 55 and 56
			5 × 10^8^	0.0022	43	
			1 × 10^9^	0.0032	54	
			1 × 10^10^	0.0040		
C_20_H_36_O_14_	1.0	2.6 × 10^5^	≥1 × 10^8^	0.0031	42	14, 54 and 55
C_5_H_10_O_5_	1.0	1.36 × 10^9^	1 × 10^9^	Evaporation > condensation	32	55, 56 and 59
			2.5 × 10^9^	0.0024	54	
			5 × 10^9^	0.0040	66	
			1 × 10^10^	0.0049		
Oleic acid	1.0	4.62 × 10^8^	1 × 10^10^	Evaporation > condensation	24	55 and 60
			2.5 × 10^10^	0.0018	39	
			5 × 10^10^	0.0029	46	
			1 × 10^11^	0.0034		

**Fig. 11:**
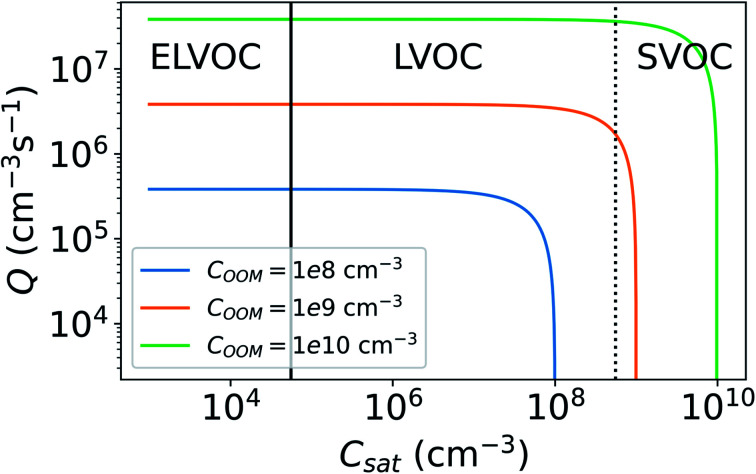
Vapor source rate (*Q*) needed to maintain a constant vapor concentration (*C*_OOM_) for different saturation concentrations (*C*_sat_). For the vapor balance equations used see eqn (4) and (7). Lines indicate whether the model oxidized organic molecule (OOM) with certain *C*_sat_ would be categorized as an extremely low volatile organic compound (ELVOC), low volatile organic compound (LVOC) or semi-volatile organic compound (SVOC) (see Sect 2.3).

The lifetime of vapors, and thus also their mass balance, is influenced by their CS_eff_ (eqn (7)). If the vapor source rate and other loss rates are the same, a vapor with smaller CS_eff_ will have a higher vapor concentration. Therefore, the CS_eff_ significantly lower than the estimated value, due to inaccurate assumptions, could lead to the misinterpretation of the results. For example, when estimating the source rate (*Q*) of vapors in a chamber study using mass balance methods with known vapor concentrations, the source rate is determined using the theoretical loss rate.^14^ If the loss rate is overestimated due to inaccurate assumptions for CS_eff_, the source rate will be overestimated. This is demonstrated in Fig. 11, which shows *Q* needed to maintain constant vapor concentrations of the model OOM (*C*_OOM_) for different *C*_sat_. *Q* has been determined based on eqn (7). We see that with increasing *C*_sat_, *Q* first appears to stay constant after reaching a certain point after which evaporation of vapor from particles becomes more and more significant and *Q* needed to maintain *C*_OOM_ steeply decreases.

Differences in the CS and CS_eff_ can also lead to differences in the rate at which vapor forms molecular clusters. If we want to describe the dynamics of atmospheric vapors and aerosol particles as accurately as possible, for example in an atmospheric model, we should account for the differences in the loss rates to particles of different vapors. The differences caused by different sizes of molecules can usually be taken into account if the chemical composition is known, but due to the lack of reported values of evaporation rates or saturation concentrations of vapors, accounting for evaporation of vapor from particles it is often difficult. Similarly, there's still uncertainty related to *α* and it is unclear whether its values could for example differ between rural and urban areas.

When determining the CS, the choice of the condensing vapor should be considered. If the CS is for example used to characterize and compare the pre-existing particle surface area, which also acts as a sink for freshly formed particles, in different locations, then staying consistent with the choice of condensing vapor and its properties is of more importance than the exact choice of the vapor species. However, if the CS is used to calculate the rate at which vapor is lost to the particles, then the choice of vapor properties is important.

If we are interested in the loss rate of vapors taking part in new particle formation, we should consider whether the vapor is SA or some other compounds such as ELVOCs and choose the properties accordingly. It should also be considered whether the condensing SA is present as pure SA molecules or in clusters with bases, such as ammonia and DMA, or water. For example, if the average cluster consists of one SA molecule and one DMA molecule, error in the CS is around 20% if properties for pure sulfuric acid molecule are used to determine the CS.

Many studies suggest that *α* = 1 and unless more specific information available it is usually the best assumption when determining the CS and CS_eff_. However, if the vapor appears to be lost at a lower rate than the CS or CS_eff_ suggests, *α* being lower than unity could be a potential explanation for this.

Finally, we recommend considering whether the CS or CS_eff_ should be used. If evaporation of the vapor of interest from particles is non-negligible, the CS_eff_ and CS are different and the CS does not describe the net loss rate of the vapor accurately. This can result in a significant error if we are for example interested in the atmospheric vapor mass balance. In most atmospheric environments, the CS and CS_eff_ for SA are likely equal due to the low volatility of SA and thus using the CS to describe the total loss rate of vapor does not result in error. However, if the considered environment has both low RH and base concentrations, it is possible that CS_eff_ is lower than CS. For organic vapors, the CS_eff_ can be considerably lower than the CS. For ELVOCs, CS ≈ CS_eff_, but for LVOC and SVOC evaporation can considerably affect the loss rate of vapor to particles. When determining the loss rate of LVOCs and SVOCs, we recommend using the CS_eff_ instead of the CS.

## 4 Conclusions

In this study, we investigate how vapor properties, such as vapor molecular mass, influence the condensation sink (CS). We also evaluate the dependency of the CS on the mass accommodation coefficient (*α*), characterizing the fraction of collisions between molecules and particles that stick. We also study the effect of evaporation on the total net vapor loss rate to particles, characterized by the effective condensation sink (CS_eff_). Often, when determining the vapor loss rate, condensing vapor is assumed to be sulfuric acid, *α* is assumed to be equal to unity and evaporation is neglected. We evaluate the uncertainty arising from these assumptions.

We determine the CS and CS_eff_ using median NPF and non-NPF day particle number size distributions from Beijing, China between January 2018 and March 2019. In addition, we use a median NPF day particle number size distribution from Hyytiälä, Finland. The differences in qualitative behavior of our results between the different size distributions are minor suggesting that our results are applicable to environments with different size distributions of background aerosol particles.

Our results show that the CS depends on the size of the vapor molecule or cluster: compounds with larger molecular mass are lost at a slower rate compared to compounds with smaller masses. For example, if a major fraction of the condensing vapor is oxidized organic compounds with large molecular masses, but their properties for CS calculation are assumed to be those of sulfuric acid, the loss rate of the condensing vapor will be overestimated. In addition, if a significant fraction of sulfuric acid is present in clusters with bases, such as dimethylamine, the CS is lower than for pure sulfuric acid molecules.

We demonstrate that if *α* differs from unity, the CS can be significantly overestimated if unity is assumed. Many studies suggest that *α* is close or equal to unity and thus this is likely not a significant source of error in reported values of CS. However, until there is a more unified agreement on values of *α* in different atmospheric environments, this element of uncertainty remains.

We show that the net loss rate of vapor can be influenced by evaporation of vapor from particles and thus the CS determined assuming irreversible condensation is an upper limit for the vapor loss rate. The CS_eff_ may be significantly lower than the CS for example for semi-volatile organic compounds. However, in most urban and rural tropospheric environments evaporation can be expected to have a little effect on the net loss rate of sulfuric acid due to the presence of stabilizing bases and water.

The investigated assumptions are shown to be able to cause significant uncertainty in the CS and CS_eff_. Overestimation of the condensation loss rate due to inaccurate assumptions can lead to inaccurate understanding of the vapor mass balance and to underestimation of the fraction of vapor molecules that forms clusters. Thus, it is important to take into consideration the properties of the condensing vapor when determining the CS. In addition, possible uncertainty in the CS due to *α* should be kept in mind. If saturation concentrations of vapors are known, it is also recommended to account for evaporation for organic vapors, when considering the net loss rate of vapor to particles. Our results can in future be applied in describing the vapor loss rates more accurately, for example in atmospheric clustering and particle formation models. Overall, our results increase the understanding of the factors influencing the vapor condensation loss rate in the atmosphere.

## Conflicts of interest

There are no conflicts of interest to declare.

## Supplementary Material
